# Extracellular vesicles secreted by primary human bronchial epithelial cells reduce *Pseudomonas aeruginosa* burden and inflammation in cystic fibrosis mouse lung

**DOI:** 10.1152/ajplung.00253.2023

**Published:** 2023-12-12

**Authors:** Sharanya Sarkar, Roxanna Barnaby, Amanda B. Nymon, Douglas J. Taatjes, Thomas J. Kelley, Bruce A. Stanton

**Affiliations:** ^1^Department of Microbiology and Immunology, Geisel School of Medicine at Dartmouth, Dartmouth College, Hanover, New Hampshire, United States; ^2^Department of Pathology and Laboratory Medicine, Center for Biomedical Shared Resources, Larner College of Medicine, University of Vermont, Burlington, Vermont, United States; ^3^Department of Genetics and Genome Sciences, Case Western Reserve University, Cleveland, Ohio, United States

**Keywords:** cystic fibrosis, extracellular vesicles, host-pathogen, Pseudomonas aeruginosa

## Abstract

Cystic fibrosis (CF) results in a reduction in the volume of airway surface liquid, increased accumulation of viscous mucus, persistent antibiotic-resistant lung infections that cause chronic inflammation, and a decline in lung function. More than 50% of adults with CF are chronically colonized by *Pseudomonas aeruginosa* (*P. aeruginosa*), the primary reason for morbidity and mortality in people with CF (pwCF). Although highly effective modulator therapy (HEMT) is an important part of disease management in CF, HEMT does not eliminate *P. aeruginosa* or lung inflammation. Thus, new treatments are required to reduce lung infection and inflammation in CF. In a previous in vitro study, we demonstrated that primary human bronchial epithelial cells (HBECs) secrete extracellular vesicles (EVs) that block the ability of *P. aeruginosa* to form biofilms by reducing the abundance of several proteins necessary for biofilm formation as well as enhancing the sensitivity of *P. aeruginosa* to β-lactam antibiotics. In this study, using a CF mouse model of *P. aeruginosa* infection, we demonstrate that intratracheal administration of EVs secreted by HBEC reduced *P. aeruginosa* lung burden and several proinflammatory cytokines including IFN-γ, TNF-α, and MIP-1β in bronchoalveolar lavage fluid (BALF), even in the absence of antibiotics. Moreover, EVs decreased neutrophils in BALF. Thus, EVs secreted by HBEC reduce the lung burden of *P. aeruginosa*, decrease inflammation, and reduce neutrophils in a CF mouse model. These results suggest that HBEC via the secretion of EVs may play an important role in the immune response to *P. aeruginosa* lung infection.

**NEW & NOTEWORTHY** Our findings show that extracellular vesicles secreted by primary human bronchial epithelial cells significantly reduce *Pseudomonas aeruginosa* burden, inflammation, and weight loss in a cystic fibrosis mouse model of infection.

## Graphical abstract

**Figure:**
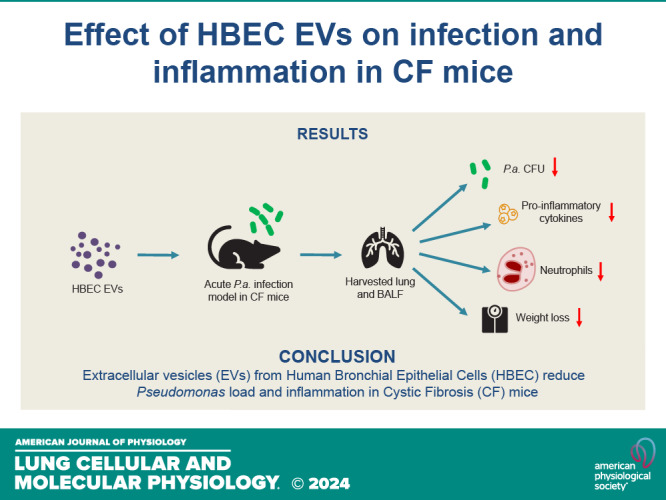

## INTRODUCTION

Cystic fibrosis (CF), the most fatal genetic disease in the Caucasian population, is a multisystem disorder affecting the lungs, gut, pancreas, liver, and kidneys. However, lung disease is the most severe, accounting for around 90% of premature deaths in people with CF (pwCF) (1). Caused by mutations in the cystic fibrosis transmembrane conductance regulator (CFTR) gene, CF is associated with a reduced volume of airway surface liquid overlying pulmonary epithelial cells, increased accumulation of viscous mucus, and entrapped pathogens (2–6). Although the CF lung is frequently infected by *Staphylococcus aureus* (*S. aureus*), *Haemophilus influenzae* (*H. influenzae*), and *Burkholderia cenocepacia* (*B. cenocepacia*), ∼50% of adult pwCF are colonized by *Pseudomonas aeruginosa* (*P. aeruginosa*) (7–12). *Pseudomonas aeruginosa* persists even in the presence of rigorous antimicrobial therapy and is associated with an increase in exacerbations, and reduced lung function, and is the primary reason for morbidity and mortality in pwCF (8, 13–17). The chronic infection triggers an intense inflammatory response that fails to eradicate the infection but perpetuates with time, further decreasing lung function (18, 19). Neutrophils and macrophages are driven to the CF lung by chemotactic signals but fail to resolve the infection. Instead, they accumulate in the lung tissue and worsen inflammation via the secretion of proteases and pro-oxidants (20, 21). High levels of neutrophil-secreted elastase trigger the activation of matrix metalloproteinase that damages airway epithelial tissue (22). CF also reduces the ability of neutrophils and macrophages to eliminate bacteria (6). In addition, macrophages in the CF airway also have reduced bactericidal activity due to the deficient production of Specialized Pro-resolving Mediators (SPM) (23–25). Although highly effective modulator therapy (HEMT) improves lung function, and decreases hospitalization in pwCF, HEMT does not eliminate bacterial lung infections or the hyperinflammatory response to chronic infections (12, 26, 27). Moreover, chronic *P. aeruginosa* infection becomes antibiotic-resistant in pwCF (13, 14, 28). Thus, new treatments are required to reduce *P. aeruginosa* infection and inflammation in CF.

Extracellular vesicles (EVs) serve a central role in intra- and inter-kingdom communication in all three domains of life, including bidirectional signaling between hosts and pathogens (29–33). This mode of cell signaling does not require direct cell-cell contact and enables host-pathogen interaction even when the bacteria reside in the mucus layer overlying lung epithelial cells, as they do in pwCF (34, 35). EVs are nanosized (10 to 1,000 nm), cell-derived, lipid bilayer-enclosed spherical entities, containing several biomolecules, including proteins, mRNA, lipids, DNAs, and microRNAs (miRNAs) (29, 36). EVs containing miRNA have been detected in several biological samples including bronchoalveolar-lavage fluid (BALF), urine, blood, and exhaled-breath condensates (37–40). miRNAs are 21- to 25-nucleotides long, noncoding RNAs that modify target-cell gene expression (41, 42). Numerous studies have demonstrated that EVs and bioengineered EVs to contain miRNA can be used to treat many diseases including cancer, but to the best of our knowledge have not been used to treat bacterial lung infections.

Previously, we demonstrated that EVs secreted by primary human bronchial epithelial cells (HBECs) deliver the miRNA let-7b-5p to *P. aeruginosa* (43) and reduced the ability of *P. aeruginosa* to form biofilms by decreasing the abundance of numerous proteins necessary for biofilm formation. In addition, let-7b-5p enhanced the sensitivity of *P. aeruginosa* to β-lactam antibiotics by inhibiting numerous genes in the β-lactam resistance pathway (43). We also determined bioinformatically that let-7b-5p is predicted to target biofilm gene orthologs in *B. cenocepacia*, another CF pathogen that quickly develops antibiotic resistance, suggesting that let-7b-5p has broad-spectrum antimicrobial activity beyond *P. aeruginosa* (43). Let-7b-5p is anti-inflammatory and is reduced in BALF of pwCF, an effect that may result, in part, in increased inflammation in the CF lung (44–47). As of 2020, there were six clinical trials involving members of the let-7 miRNA family to treat a variety of diseases such as diabetes, obesity, and cancer (45, 48). However, to the best of our knowledge, there is no clinical trial using let-7b-5p to treat bacterial infections. Because experiments in our previous work were performed in vitro, in this study, we performed experiments to examine the in vivo effects of HBEC EVs, naturally containing let-7b-5p, on *P. aeruginosa* infection and the inflammatory responses in CF mice using the agar bead model of infection (49, 50). Although this is not a model of chronic lung infection, it does recapitulate the CF phenotype of an aggravated inflammatory response, especially the enhanced neutrophil recruitment pattern (19, 49–51). Our data demonstrate that HBEC EVs, even in the absence of any antibiotic, decreased *P. aeruginosa* in the lungs, as well as the infection-related loss in body weight, and decreased neutrophils and several key proinflammatory cytokines in the BALF. These data, coupled with our published in vitro data, demonstrate that HBEC EVs significantly reduce *P. aeruginosa* lung infection and inflammation in CF.

## MATERIALS AND METHODS

### Culture of Human Bronchial Epithelial Cells

Primary bronchial epithelial cells [HBECs, wild type (WT), or CF (Phe508del homozygous)], obtained from Dr. Scott Randell (University of North Carolina, Chapel Hill, NC), were cultured as previously described (52). Briefly, the cells (*passages 4*–*8*) were grown in BronchiaLife basal medium (Lifeline Cell Technology, Frederick, MD) at 37°C (5% CO_2_) supplemented with BronchiaLife B/T LifeFactors Kit (Lifeline), 10,000 U/mL penicillin, and 10,000 μg/mL streptomycin (Sigma Aldrich, St. Louis, MO). Cells were tested routinely for mycoplasma contamination. Before EV collection, cells (96%–98% viability as determined by Bio-Rad TC10 Automated Cell Counter) were seeded at 2 × 10^6^ cells per T175 and grown to confluence while replenishing the growth medium every 2 days.

### IRB Statement

Deidentified primary human bronchial epithelial cells from six CF donors (CF-HBECs, Phe508del homozygous) were obtained from Dr. Scott Randell (University of North Carolina, Chapel Hill, NC) and cultured as described previously (52). Since the cells were acquired from discarded tissue and comprised no patient identifiers, their use in this study was not considered as human subject research by the Dartmouth Committee for the Protection of Human Subjects.

### EV Isolation and Characterization

**Figure 1.**
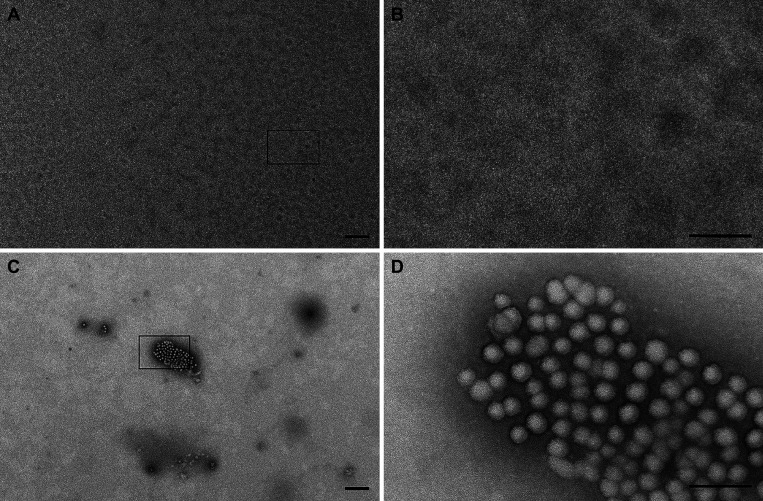
Transmission electron micrographs show the lack of identifiable vesicles in the process control under low magnification (×6,000; *A*) and high magnification (×50,000; *B*). Transmission electron micrographs show the presence of human bronchial epithelial cell (HBEC) extracellular vesicles (EVs) under low magnification (×6,000; *C*) and high magnification (×50,000; *D*). The boxes in *A* and *C* indicate the region presented in *B* and *D*, respectively. Scale bars represent 500 nm and 100 nm for the low and high magnifications, respectively.

For the isolation and characterization of EVs, media was removed from the T175, washed with PBS, and replaced with the basal media lacking supplements (and antibiotics) 24 h before EVs were isolated. Recommendations outlined by the International Society of Extracellular Vesicles (ISEV) were followed for isolation and characterization of EVs (37). We isolated the EVs from the supplement-free supernatants of confluent HBEC cultures (*passages 4*–*8*), using ExoQuick-TC EV isolation kit (#EXOTC50A-1, System Biosciences, Palo Alto, CA) as described previously (43, 53). Briefly, conditioned media was separated from the cells and debris by centrifugation at 3,000 *g*, 15 min, followed by concentration with 30Kd Amicon Ultra Centrifugal Filter (Millipore, Billerica, MA). The concentrate was mixed with 50–80 μL of ExoQuick-TC polymer, and following overnight incubation at 4°C, EVs were isolated by centrifugation (1,500 *g*, 30 min). Media not conditioned by cells was subjected to the same isolation process and was termed process control to ensure that no contaminant was introduced during the isolation process. To standardize dosing of EVs in mice, Nanoparticle Tracking Analysis (NTA 3.4, NanoSight NS300, Malvern Panalytical Ltd., Malvern, UK) was used to quantify particle number under the following settings: screen gain of 1, camera level of 15, process screen gain of 10, detection threshold of 5, continuous syringe pump flow of 30, dilution of 1:400, and three 30 s acquisitions. To ensure that the isolated particles were EVs, their protein content was analyzed according to ISEV guidelines (37), including assessment for the presence of exosomal markers HSP-70 and TSG-101, and the absence of nonexosomal marker calnexin by Western blot, as shown in our previous study (43). In addition, negative staining electron microscopy confirmed the presence of EVs isolated from conditioned media and absent in process control (Fig. 1).

### Electron Microscopy of EV

EVs were imaged by negative staining transmission electron microscopy. Ten microliters of each sample were incubated for 2 min on a freshly glow discharged 200-mesh nickel grid, followed by wicking of the solution with filter paper, and rinsing on seven sequential drops of Millipore filtered distilled water. The still moist grids were then touched to one drop of NanoW tungsten stain (Ted Pella; Product No. 09S432, Lot No. 14702), the excess was wicked off immediately with filter paper before repeating this step on a second drop of Nano-W, which was left for 1 min before being wicked off and allowed to dry. The stained grids were imaged at 80 kV in a JEOL 1400 transmission electron microscope (JEOL Inc., Danvers, MA), and digital images were acquired in TIFF with an AMT XR11 digital camera (AMT, Woburn, MA).

For measurement of EVs by electron microscopy, images were opened in MetaMorph Offline image analysis software (v.7.8.0.0; Molecular Devices LLC, San Jose CA), and the diameters of 24 vesicles were determined by drawing a measuring line from one side of the vesicle to the other. The average diameter was then calculated from these measurements. Image acquisition and EV diameter measurements were performed by an investigator blinded to the sample composition.

### Preparation of Agarose-Laden Beads

Agarose-laden beads to embed *P. aeruginosa* were prepared as described previously (51). Briefly, five colonies of a clinical *P. aeruginosa* isolate, labeled with mCherry (mCH PA M57-15) and grown overnight on a gentamycin/LB agar were inoculated in 25 mL of LB broth and incubated at 37°C with shaking (200 rpm), for 20 h. It was then 10-fold diluted to attain an absorbance (OD 600 nm) of ∼0.3. Twenty-three milliliters of the mCH PAM57-15/agarose/PBS mixture were added to a beaker containing 250 mL of sterile mineral oil and set on medium-high stir. After 10 min, the bead/oil mixture was added to 15 mL of prewarmed 0.5% sodium deoxycholate/PBS and centrifuged to separate the beads from the oil layer. Following a second wash, the beads were transferred to fresh conical tubes containing 20 mL of PBS and washed four times. The beads were then assessed by imaging under the red channel. Following dilution plating on gentamycin/LB agar plates and colony-forming unit (CFU)/mL evaluation, the bead/PBS mixture was diluted to achieve a concentration of 25,000 CFU per 50 µL.

### *Pseudomonas aeruginosa* Inoculation in Mice

F508del/F508del, gut-corrected mice, on a C57Bl/6 background were used for all studies (54). All animal protocols were approved by the Institutional Animal Care and Use Committee (IACUC) of Case Western Reserve University. Both male and female mice, 10–12 wk old, were inoculated with agar beads impregnated with 10^5^ CFU of the CF clinical strain mCH PA M57-15 and 10^8^ HBEC EVs (standardized using particle number from Nanoparticle Tracking Analysis) secreted by WT or CF HBEC or the same volume of control (saline or process control) using a micro-endoscopy system (Polydiagnost, Pfaffenhofen, Germany) (Table 1). The inoculum was delivered to the right lungs of the mice because our preliminary experiments indicated that this method yields higher inoculation efficiencies while reducing the variability of infection. The mice were placed on warming pads inside sterile cages and monitored until they emerged from the isofluorane-induced anesthesia. The condition of inoculated mice was evaluated daily up to the day of sample collection, as described previously (51, 55).

**Table 1.** HBEC donors and number of mice exposed to EVs from each donor

EV Donor	Number of Mice
55J—WT EV	5
32I—WT EV	4
42I—WT EV	4
32G—CF EV	4
29H—CF EV	4
36J—CF EV	6
Control—saline	5
Control—process control	9

Extracellular vesicle (EV) group [wild-type cystic fibrosis transmembrane conductance regulator (CFTR) (WT EV) and cystic fibrosis (CF) donors (CF EV)]. Process control (unconditioned cell culture media run through the EV isolation process). HBEC, human bronchial epithelial cell.

Three days after the infection was initiated mice were euthanized by CO_2_ inhalation. After disinfection with 70% ethanol, a surgical cut was made along the midline to uncover the thoracic cage, and then the diaphragm was incised to expose the lungs. Bronchoalveolar lavage fluid (BALF) was collected and both right and left lungs were homogenized individually and cultured for *P. aeruginosa* growth (reported in CFU/mL). The total lung burden (in CFU/mL) was calculated by totaling the number of bacteria recovered from three regions—the BALF, the left lung homogenate, and the right lung homogenate. A blinded methodology was used to evaluate total white blood cells from 10 µL of BALF using a hemocytometer, whereas cell differentials were estimated from 200 µL of BALF using Giemsa/Miller staining. Cytokines were measured using a 32-plex Luminex platform on *day 3* (peak of the inflammatory response). All mouse experiments and sample acquisition were performed by an investigator blinded to the treatment.

### Statistical Analysis

The determination of sample size was supported by conducting a power analysis using a two-sided, two-sample unequal-variance *t* test. As described previously (51), it was determined that for α = 0.02, a sample size of 10 in each group was enough to achieve >90% power to identify differences in recruitment of inflammatory cells. To reduce experimental bias, all differential cell counts and cytokine analyses were performed by personnel blinded to experimental groups. Male and female mice were both included to account for any possible gender differences in treatments. However, no sex differences were noted by performing interaction analyses (56), so we combined the data from both sexes, as indicated in the results. In addition, using linear mixed-effects models, there was no significant difference between saline and process control exposed mice across different batches, thus, the data were combined. Importantly, this analysis demonstrated that the cell culture media or EV isolation procedure did not contain anything that had an effect on the response of mice to *P. aeruginosa* compared with saline control.

Data were analyzed using GraphPad Prism (v.9.1.2; San Diego, CA) and the *R* software environment for statistical computing and graphics version 4.1.0 (57). Since there was no statistically significant difference in total CFU burden, neutrophils, immune cells, cytokines, or percent weight loss between mice receiving saline or process control, the data were combined. Similarly, there was no statistically significant difference between the effect of WT EVs or CF EVs, thus the data were combined from mice receiving WT EVs or CF EVs. Following this, linear mixed-effects models were used to test statistical significance between control and EV-exposed mice for all parameters (CFU burden, cytokines, immune cells, and weight loss) while accounting for potential batch effects. The *R* package nlme (v.3.1-162) was used to calculate *P* values from linear mixed-effect models (58). The cytokine heatmap figure was generated using the ComplexHeatmap package (v.2.8.0) (59).

## RESULTS

### Isolation and Characterization of HBEC EVs

We isolated and characterized HBEC EVs (secreted by wild-type and CF donors) following the recommendations of the International Society of Extracellular Vesicles (37). Unconditioned cell culture media (process control) as well as HBEC-conditioned media were run through the ExoQuick-TC isolation process to isolate EVs. Transmission electron microscopy demonstrated that the process control lacked identifiable EVs (Fig. 1, *A* and *B*), whereas the EVs secreted by HBEC were 56.8 nm in diameter (Fig. 1, *C* and *D*). In a previous study using ExoQuick-TC to isolate EVs, we demonstrated by Western blot that the EVs isolated from HBECs were enriched in two key EV markers, HSP70 and TSG101, but not the non-EV calnexin (43). In that study, we also validated that HBEC EVs fuse with *P. aeruginosa* and deliver their cargo into the bacterial cells (43).

### HBEC EVs Reduce *Pseudomonas* Burden in CF Mice

**Figure 2.**
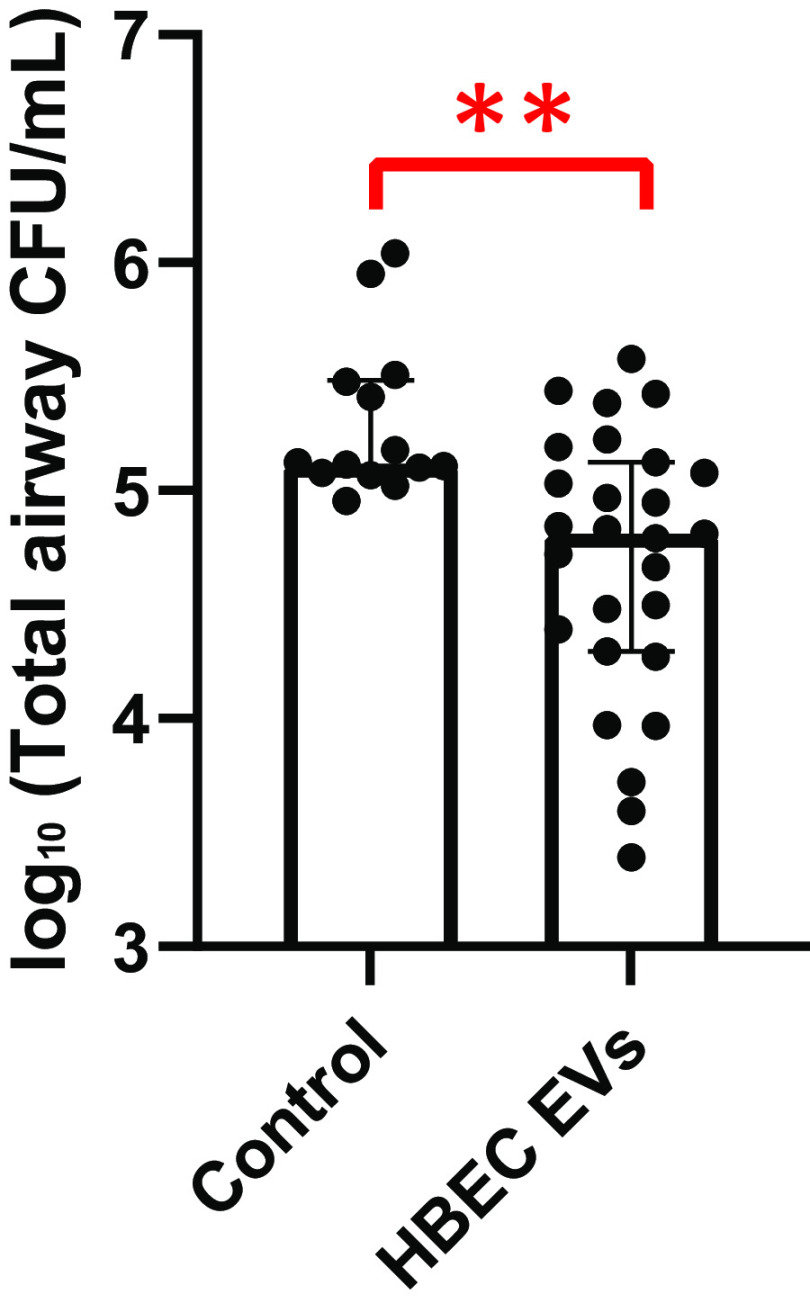
Human bronchial epithelial cell (HBEC) extracellular vesicles (EVs) reduced total lung *Pseudomonas aeruginosa* counts (CFU/mL) in CF mice, compared with the control group. The control group included mice receiving saline or process control. There was no difference between saline and process control results, thus the data were combined. Sex of the animal was not a modifier of the response to treatment, so data from male and female mice were combined. Linear mixed-effects models were used to account for potential batch effects to test statistical significance between control and EV groups. Each data point represents one cystic fibrosis (CF) mouse. **P < 0.01; *n* = 14–27 mice/group; median ± interquartile range depicted. Data were compiled from 4 independent experiments. CFU, colony-forming units.

To assess whether HBEC EVs reduced the burden of a CF clinical isolate of *P. aeruginosa* in vivo, CF mice were inoculated with *P. aeruginosa*-impregnated agarose beads via an endoscope inserted into the trachea along with either HBEC EVs or saline or process control. Three days later, lung tissue and BALF were collected for analysis of *P. aeruginosa* colony-forming units, neutrophils, cytokines, and chemokines. HBEC EVs reduced *P. aeruginosa* colony-forming units (CFUs) by 66.7% (0.6 log_10_), as compared with the control group (Fig. 2). Although female CF mice had a higher median bacterial load in both the control and EV treatment groups, as compared with male CF mice, interaction analyses revealed that the sex of the animal did not act as a modifier of the bacterial load response to the administered treatment (*P* value = 0.55). Consequently, we were able to pool data from both the sexes. Furthermore, our observation was consistent with previous studies showing that females with CF tend to have a higher bacterial burden than males with CF (60).

### HBEC EVs Reduce Proinflammatory Cytokine Levels in CF Mice

**Figure 3.**
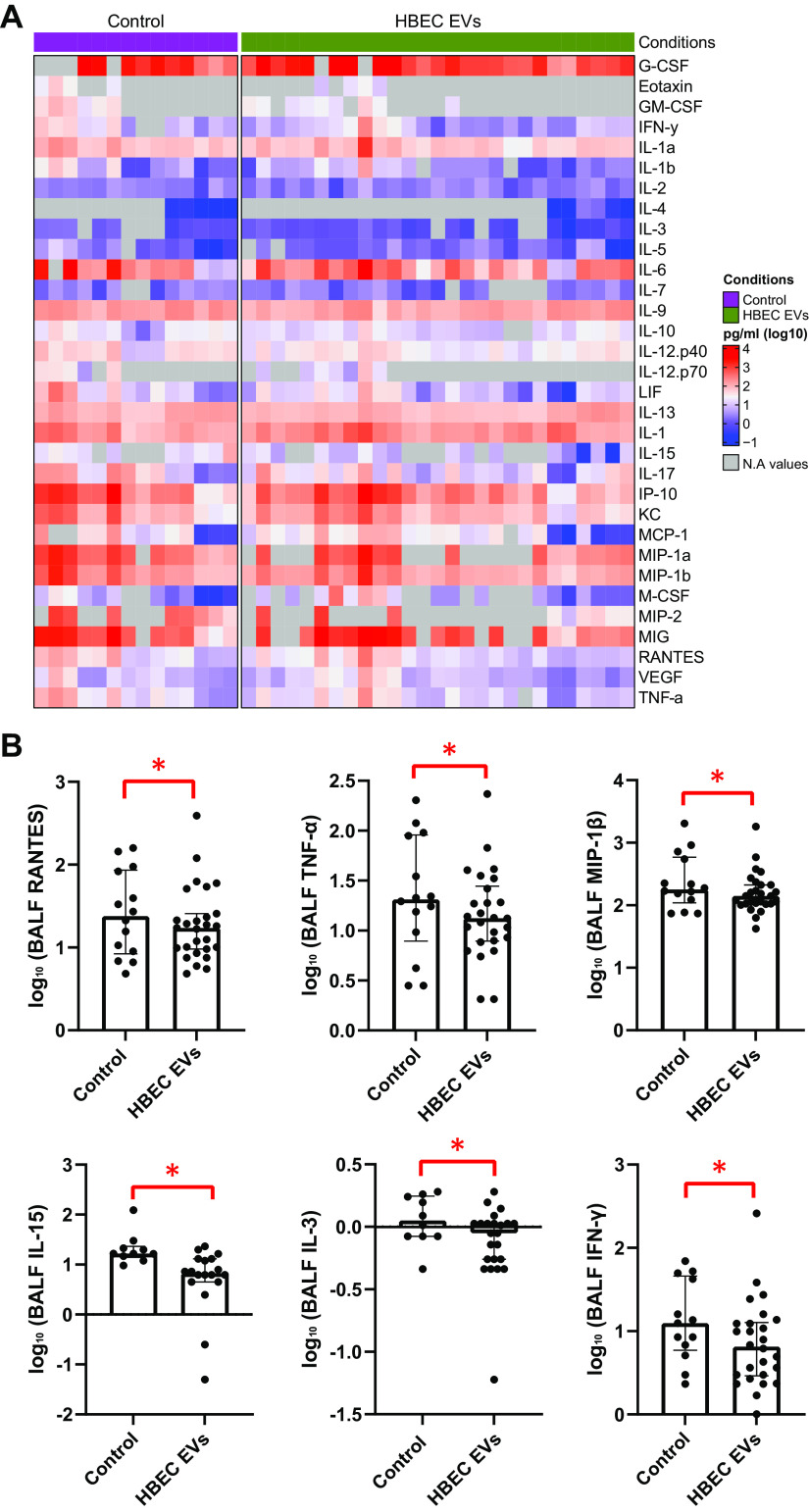
*A*: heatmap depicting the log10 transformed secreted cytokine concentrations (pg/mL) of the 32 cytokines in bronchoalveolar lavage fluid (BALF) in the control and human bronchial epithelial cell (HBEC) extracellular vesicle (EV) groups. The gray boxes denote cytokines that were below the detection level of the multiplex assay. Blue indicates cytokine concentrations close to 0.1 pg/mL, whereas red indicates cytokine concentrations ∼10,000 pg/mL. *B*: HBEC EVs significantly reduced several key proinflammatory cytokines including Regulated upon Activation, Normal T Cell Expressed and Presumably Secreted: RANTES, TNF-α, Macrophage Inflammatory Protein: MIP-1β, IL-15, IL-3, and IFN-γ in the BALF of cystic fibrosis (CF) mice as compared with control mice. N for control and EV- treated mice varied for the cytokines, since some mice had cytokine levels beyond detection level of the multiplex platform. Controls comprised mice receiving saline or process control: there was no significant difference between saline or process control, thus, the data were combined. Sex of the animal was not a modifier of the response to treatment, so data from male and female mice were combined. Linear mixed-effects models were used to account for potential batch effects to test statistical significance. Each data point represents one CF mouse. *P < 0.05; *n* = 10–27 mice/group; median ± interquartile range depicted. Data were compiled from 4 independent experiments.

Since HBEC EVs reduced the bacterial burden in CF mice, we assessed the impact of HBEC EVs on inflammation by measuring the levels of cytokines in BALF using Luminex multi-plex (Millipore Mouse 32-Plex Kit) technology (Fig. 3*A*). We found that among the 32 cytokines measured, there were eight proinflammatory cytokines that were significantly reduced in CF mice exposed to HBEC EV compared with control—namely, Granulocyte-Macrophage Colony-Stimulating Factor: GM-CSF (*P* = 0.04), IFN-γ (*P* = 0.03), IL-3 (*P* = 0.01), IL-15 (*P* = 0.01), Macrophage Inflammatory Protein: MIP-1β (*P* = 0.03), MIP-2 (*P* = 0.01), Regulated upon Activation, Normal T Cell Expressed and Presumably Secreted: RANTES (*P* = 0.03), and TNF-α (*P* = 0.04). We were particularly interested in examining the data distribution for six cytokines that did not have out-of-range (OOR) values for the majority of the samples including RANTES, TNF-α, MIP-1β, IL-15, IL-3, and IFN-γ (Fig. 3*B*). These proinflammatory cytokines are biologically relevant in CF: *1*) RANTES is a determinant of the production of mediators dependent on STAT1 activation, a pathway that is deficient in CF epithelial cells (61), *2*) TNF-α is a determinant of the production of other proinflammatory cytokines due to its paracrine effects, *3*) MIP-1β is a proinflammatory cytokine that recruits macrophages and T-cells to the infection site (62), *4*) IL-15 is induced by TNF-α and IFN-γ and promotes a proinflammatory microenvironment (63), *5*) IL-3 has been implicated in acute lung injury in the mouse model (64), and *6*) IFN-γ induces RANTES in macrophages via IRF-1 (65). Cytokine levels in BALF were not determined by the sex of mice, as validated by interaction analyses. Taken together, the data demonstrate that HBEC EVs significantly downregulated several key and biologically relevant proinflammatory cytokines in the BALF of CF mice during acute *P. aeruginosa* infection.

### HBEC EVs Decrease Neutrophil Recruitment in CF Mice

**Figure 4.**
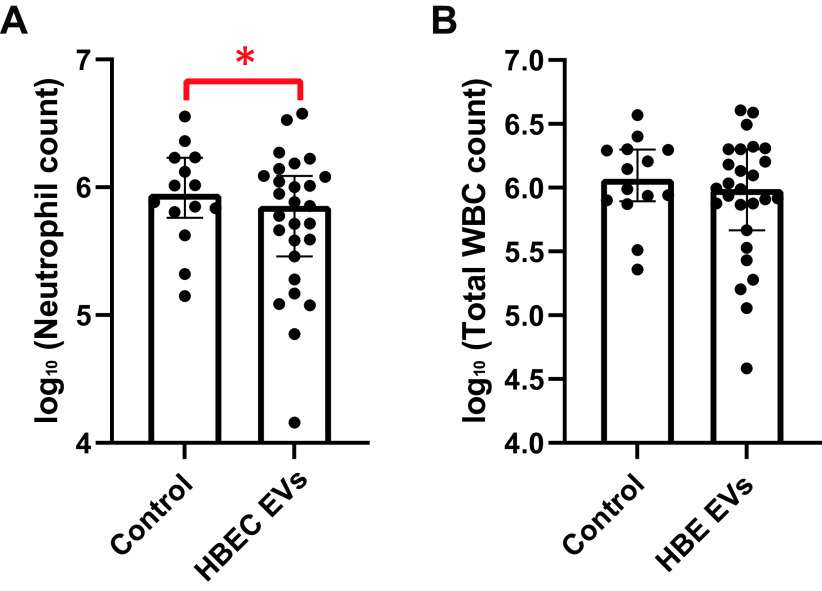
*A*: human bronchial epithelial cell (HBEC) extracellular vesicles (EVs) significantly reduced neutrophils in the bronchoalveolar-lavage fluid (BALF) of cystic fibrosis (CF) mice compared with the control group. *B*: total WBC count showed a tendency to decrease in the HBEC EV group but did not achieve statistical significance. The control group comprised mice receiving saline or process control (there was no statistical difference between the saline and process control data). Sex of the animal was not a modifier of the response to treatment, so data from male and female mice were combined. Each data point in the figures represents one CF mouse. To test statistical significance, linear mixed-effects models were used to account for potential batch effects. *P < 0.05; Control *n* = 14 mice, HBEC EVs *n* = 27 mice; median ± interquartile range depicted. Data were compiled from 4 independent experiments.

Because HBEC EVs reduced *P. aeruginosa* infection and cytokines in BALF, studies were conducted to examine the effect of HBEC EVs on immune cells in BALF. Compared with the control group, HBEC EVs significantly reduced neutrophil counts (Fig. 4*A*). There was a trend for EVs to reduce the total WBC count, but it did not reach statistical significance (Fig. 4*B*). CF mice that received HBEC EVs had reduced neutrophil counts in the BALF in response to *P. aeruginosa*, consistent with the fact that reduced levels of proinflammatory cytokines lead to reduced recruitment of neutrophils.

### HBEC EVs Minimized Weight Loss in Infected CF Mice

**Figure 5.**
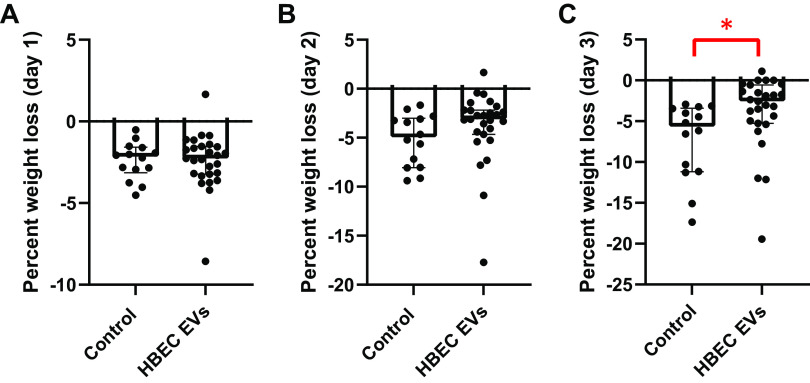
*A*: percent weight loss on *day 1*, where both control and human bronchial epithelial cell (HBEC) extracellular vesicle (EV) groups experience similar weight loss. *B*: percent weight loss on *day 2*, where mice receiving HBEC EVs have attenuated weight loss, although the attenuation is not statistically significant as compared with the control group. *C*: percent weight loss on *day 3*, where the HBEC EV group has significantly reduced percent weight loss as compared with the control group. The control group comprised mice receiving saline or process control. Sex of the animal was not a modifier of the response to treatment, so data from male and female mice were combined. Each data point represents 1 cystic fibrosis (CF) mouse. To test statistical significance, linear mixed-effects models were used to account for potential batch effects. *P < 0.05; Control *n* = 14 mice, HBEC EVs *n* = 27 mice; median ± interquartile range depicted. Data were compiled from 4 independent experiments.

Weight loss due to infection is an informative marker of efficacy of therapy in CF mice to *P. aeruginosa* (51). Both control and HBEC EV mice lost a similar amount of weight on *days 1* and *2* after exposure to *P. aeruginosa* (Fig. 5, *A* and *B*). However, on *day 3*, the weight loss was attenuated in mice receiving HBEC EVs, an observation consistent with the reduction in CFU and inflammation (Fig. 5*C*).

## DISCUSSION

Here, we report that EVs secreted by primary HBECs reduce *P. aeruginosa* burden and inflammation in the CF mouse lung. We demonstrated that HBEC EVs significantly reduced total airway bacterial load (CFU/mL), proinflammatory cytokine levels, and neutrophil counts in the BALF, and reduced inflammation-associated weight loss in CF mice. Moreover, there were no significant differences in the response of male and female mice to EVs. The results in this report suggest that the secretion of EVs by HBEC is a mechanism that the host may use to respond to infection, thereby minimizing inflammation and neutrophils that are known to damage the lungs and reduce lung function (e.g., forced expiratory volume in one second: FEV_1_).

It is interesting to note that human mesenchymal stromal cells (hMSCs) also attenuate both *P. aeruginosa* burden and inflammation in a sublethal CF mouse model of infection (66). Infected mice treated with hMSCs also had reduced proinflammatory cytokines, diminished severity of gross lung pathology, and weight loss (66). Another study revealed that EVs from MSCs were effective in decreasing *P. aeruginosa* in a murine model of pneumonia (67) and that the beneficial effects were attributed to miRNA-466 in the MSC EVs (67). In addition, MSC EVs also reduced the bacterial burden in a pneumonia model of *E. coli* infection (68, 69) and in ex vivo human lungs infected with *E. coli* (70, 71). A recent study has shown that HBEC EVs inhibit pulmonary fibrosis in vivo via inhibiting TGF-β-WNT cross talk (72). However, to the best of our knowledge, no previous study has demonstrated that EVs secreted by HBEC abate *P. aeruginosa* burden or lung inflammation in a CF model of infection. Taken together with our previous study demonstrating in vitro that HBEC EVs and let-7b-5p reduce *P. aeruginosa* biofilm formation and antibiotic resistance, the in vivo mouse studies suggest that bioengineering EVs to contain let-7b and antibiotics may be an effective treatment to eliminate *P. aeruginosa* lung infections and hyperinflammation in pwCF.

The effect of EVs on reducing the bacterial burden and the immune response of CF mice is likely to be multifactorial. First, it is likely that the EVs secreted by HBEC enhance the ability of lung macrophages to phagocytose and kill *P. aeruginosa* since MSC EVs enhance phagocytosis and the ability to kill bacteria by macrophages (69, 73–75). Second, we also consider the possibility that HBEC EVs increase the secretion of antimicrobial peptides by HBEC, thereby indirectly reducing the bacterial burden. Additional studies, beyond the scope of the present study, are required to elucidate the mechanisms whereby HBEC EVs reduce *P. aeruginosa* lung infection and inflammation in the CF mouse.

Our study has several advantages, including *1*) The use of primary HBEC from multiple donors, which is more representative of the human population than immortalized cell lines isolated from a single donor (76), *2*) The use of process control (unconditioned media passed through the EV isolation process) for possible artifacts that may be introduced in the HBEC culture media and/or EV isolation process that could have confounded the results. It is important to note that the results from mice exposed to process control or saline control were not statistically different, consistent with the conclusion that the EV isolation procedure did not introduce an artifact, and *3*) Our examination of the impact of EVs on weight loss makes this study even more significant since it agrees with the existing literature that there is a strong correlation between body mass index (BMI) and severity of lung disease in patients with CF (77–81).

There are a few limitations of our study. First, HBEC EVs did not completely eliminate *P. aeruginosa* infection, raising concern about the clinical utility of EVs in treating antibiotic-resistant infections. However, because in our previous study, we demonstrated that let-7b-5p in HBEC EVs in combination with half the minimal inhibitory concentration (MIC) of aztreonam blocked antibiotic-resistant biofilm formation and increased the aztreonam sensitivity of planktonic *P. aeruginosa* in vitro (43), we consider it likely that bioengineered EVs or nanoparticles containing let-7b-5p and aztreonam will be more effective in eliminating *P. aeruginosa* burden and inflammation in the CF mouse. In addition, we have also shown that EVs secreted by HBEC increase the sensitivity of *P. aeruginosa* to ciprofloxacin, an antibiotic like aztreonam, that is used in the clinic to treat pwCF (82). Moreover, the effect of EVs to reduce inflammation and neutrophils in BALF, even in the absence of any antibiotic, would be expected to reduce lung damage caused by *P. aeruginosa* (44–46). Another potential limitation of the current study is that the CF mice used in this study are not a perfect model to study chronic bacterial infections since the agarose bead model can only be utilized for up to a period of 10 days (51). In addition, the CF mouse does not recapitulate all of the CF phenotype in humans (83). Despite not being a model of chronic infection, these mice demonstrate the classic, neutrophil-dominated CF inflammatory response (19) and permit the evaluation of potential therapeutic interventions (51). Although CF ferrets and rats are excellent chronic infection models (84, 85), performing experiments on them is beyond the scope of this study and a potential future direction.

In addition, we acknowledge that the CF lung typically has a polymicrobial infection, comprising key species such as *P. aeruginosa*, *S. aureus, H. influenzae,* and *B. cenocepacia* (9). Since around 50% of adult pwCF are colonized by *P. aeruginosa*, our current study focused on *P. aeruginosa.* However, our previous study has shown that let-7b-5p is also bioinformatically predicted to target corresponding biofilm and antibiotic-resistant gene orthologs in *B. cenocepacia* (43). Therefore, additional experiments beyond the scope of this study are needed to elucidate the extent to which HBEC EVs and let-7b-5p can decrease the burden of other CF pathogens in the mouse model.

Finally, there is a clinical gap that needs to be addressed before HBEC EVs can be translated to the clinic. HBEC EVs are likely to be immunogenic, thus, synthetic nanoparticles or EVs from other sources such as MSCs or induced pluripotent stem cells (iPSC) can be bioengineered to contain let-7b-5p and antibiotics and tested in the CF mouse. Given that both MSCs and their EVs have been found to be safe and nontoxic in clinical trials (41, 86, 87), including in pwCF (88), the studies in this report provide solid ground for the development of MSC EV-based antimicrobials.

In summary, our data demonstrate that EVs from HBEC reduce both *P. aeruginosa* infection and inflammation in CF mice and provide proof-of-concept that EVs and/or nanoparticles, in combination with key miRNAs and antibiotics used in the CF clinic, could be pursued as a potential therapeutic for pwCF to reduce and/or eliminate chronic infection and inflammation.

## DATA AVAILABILITY

Data will be made available upon reasonable request.

## GRANTS

This work was supported by funding from the Cystic Fibrosis Foundation (CFF) under Grants STANTO19G0, STANTO20P0, STANTO23R0, and STANTO19R0 (to B.A.S.), the National Institutes of Health under Grants P30 DK117469 and R01 HL151385 (to B.A.S), and the Flatley Foundation. This work was supported by CFF RDP R447-CR11 under Grant DRUMM15R0/CFF. Mouse studies were supported by the CF Mouse Resource Center at Case Western Reserve University under Grant CFF HODGES19R1.

## DISCLAIMERS

The funders had no role in study design, data collection and analysis, decision to publish, or preparation of the manuscript.

## DISCLOSURES

No conflicts of interest, financial or otherwise, are declared by the authors.

## AUTHOR CONTRIBUTIONS

S.S., R.B., A.B.N., T.J.K., and B.A.S. conceived and designed research; S.S., R.B., D.J.T., and T.J.K. performed experiments; S.S. analyzed data; S.S., R.B., A.B.N., T.J.K., and B.A.S. interpreted results of experiments; S.S. prepared figures; S.S. and B.A.S. drafted manuscript; S.S., R.B., A.B.N., D.J.T., T.J.K., and B.A.S. edited and revised manuscript; S.S., R.B., A.B.N., D.J.T., T.J.K., and B.A.S. approved final version of manuscript.
